# Preserflo MicroShunt versus Trabeculectomy: Efficacy and Surgical Success within a Heterogenous Patient Cohort

**DOI:** 10.3390/life14091171

**Published:** 2024-09-17

**Authors:** Lynn Anna Barbara Zweifel, Jens Julian Storp, Friederike Elisabeth Vietmeier, Moritz Fabian Danzer, Ralph-Laurent Merté, Nicole Eter, Viktoria Constanze Brücher

**Affiliations:** 1 Department of Ophthalmology, University of Muenster Medical Center, 48149 Muenster, Germany; jens.storp@ukmuenster.de (J.J.S.); friederikeelisabeth.vietmeier@ukmuenster.de (F.E.V.); ralph-laurent.merte@ukmuenster.de (R.-L.M.); nicole.eter@ukmuenster.de (N.E.); viktoria.bruecher@ukmuenster.de (V.C.B.); 2 Institute of Biostatistics and Clinical Research, University of Muenster, 48149 Muenster, Germany; moritzfabian.danzer@ukmuenster.de

**Keywords:** glaucoma, MIGS, LIGS, microinvasive glaucoma surgery, TE, IOP, real-world, PEX, POAG

## Abstract

To compare success rates of trabeculectomy (TE) and Preserflo MicroShunt (PMS) in heterogenous glaucoma cohorts with regards to different pre- and postoperative therapeutic regimens. Data of 187 glaucoma patients who either received TE (73 eyes) or PMS implantation (114 eyes) between January 2018 and December 2022 were retrospectively evaluated. Surgical success and failure rates were analyzed within six months of follow-up. Intraocular pressure (IOP) development over the course of follow-up was compared between both groups. Tertiary outcome measures were best-corrected visual acuity (BCVA), number and type of medications, frequency of postoperative complications and revision surgeries. Outcome measures underwent additional assessment based on subgroup categorizations, and failure time hazard ratios were computed. The success rates were comparable between both procedures (TE: 54.1%, PMS: 60.0%; *p* = 0.17). Both procedures showed significant IOP reduction (*p* < 0.01); however, overall IOP reduction was greater in the TE group than in the PMS group (TE: Reduction by 12 mmHg (188.9%), PMS: Reduction by 7 mmHg (51.3%); *p* = 0.01). The number of topical medications decreased significantly in both groups over the course of follow-up (TE: 4 to 0, PMS: 3 to 0; *p* < 0.01). While the number of complications and revision surgeries were similar in both groups, the time interval until the first revision surgery within the TE group was significantly shorter (TE: 13.5 d, PMS: 163 d; *p* = 0.01) than within the PMS group. No difference could be detected between TE and PMS with regard to the follow-up regimen. In particular, there was no significant difference in the need for 5-floururacil injections postoperatively (*p* = 0.29). Less invasive glaucoma surgery with the PMS appeared comparable to the TE within a heterogenous glaucoma cohort with regards to IOP development and freedom from medication.

## 1. Introduction

Glaucoma is a progressive disease that can lead to irreversible vision loss if left untreated. Elevated intraocular pressure (IOP) is a key risk factor for glaucoma progression [1,2], and various treatment options have been developed to counter the effect of elevated IOP on retinal tissue. Aside from topical medication and laser surgery, surgical procedures have been established and integrated into the vast field of glaucoma treatment options [3].

Trabeculectomy (TE) remains the gold standard for IOP-lowering surgery. Medicare claims data and surveys of the American Glaucoma Society membership show that microinvasive glaucoma surgeries (MIGS) are increasingly being utilized as an alternative to TE by promising a safe and efficient IOP reduction technique that can replace current practices [4].

By creating an anatomical link between the anterior chamber and the subconjunctival space, the Preserflo MicroShunt^®^ (PMS) (Santen, Miami, FL, USA) is a biocompatible subconjunctival ab externo device, which, unlike TE, requires no scleral flap or sutures and is therefore considered a “less invasive glaucoma surgery” (LIGS) implant. LIGS devices are supposed to offer shorter procedure times, a quicker recovery and fewer complications in comparison to traditional penetrating glaucoma surgery, while at the same time effectively reducing the IOP in the long-term, irrespective of glaucoma subtype [5].

Over the past few years, there has been a growing interest in comparing TE and PMS. This is especially true since the number of LIGS and MIGS procedures has increased during the past decade [3,4]. 

Previous studies have focused primarily on PMS in primary open-angle glaucoma (POAG) [6,7,8,9], but there is a lack of long-term studies comparing TE and PMS within a real-world setting. 

In this paper, the surgical methods TE and PMS are compared in accordance with their success rates and IOP development in a heterogenous glaucoma cohort. In addition, the postoperative development of TE patients and PMS patients is evaluated with regards to disease severity and type of glaucoma.

## 2. Materials and Methods

This retrospective, monocentric study was conducted in compliance with the ethical standards issued by the ethics committee of the Medical Association of Westfalen-Lippe, Germany, and the University of Münster, Germany. The study adhered to the principles of the Declaration of Helsinki and its later amendments. 

The retrospective study included data from patients who underwent either TE or PMS implantation between 1 January 2018 and 31 December 2022. The digital documentation systems FIDUS (Ärzteservice Wente GmbH, Darmstadt, Germany) and ORBIS (Dedalus Healthcare GmbH, Bonn, Germany) provided the electronic patient records from which the data were gathered.

### 2.1. Surgical Procedure

In order to prevent conjunctival hyperemia on the day of surgery, our clinic’s patients ceased using any anti-glaucomatous eye drops four weeks before TE or PMS implantation where medically justifiable. Instead, they received oral acetazolamide for four weeks. In the case of advanced glaucoma or topically unadjustable intraocular pressure, a change in pressure-lowering therapy was dispensed with. In both cases, corticoid eye drops were administered three days prior to surgery. Then, to lessen the pre-to-postoperative pressure gradient, patients received mannitol (250 mg) and acetazolamide (500 mg) intravenously 2–4 h prior to the procedure. The surgical steps in TE have been described in detail elsewhere [10]. The following passage is therefore limited to an overview of the novel PMS system [11,12]. In short, after preparing the subconjunctival space and the forming of a scleral buckle, sponges are inserted into the conjunctival flap and mitomycin-C (MMC) 0.2 mg/mL is given to the exposed sclera for three minutes following the dissection of the conjunctiva and Tenon’s capsule. Using a 1 mm lance, a 2 mm deep scleral tunnel is made after further washing with a balanced salt solution. The anterior chamber is then reached by passing a 25-gauge needle down this tract, creating a tunnel that extends 3.5–4 mm from the limbus to the subconjunctival pocket. With its tip extending about 2 mm into the anterior chamber, the microshunt is placed ab externo into the tunnel, keeping its wings inside the scleral pocket. Tenon’s capsule and conjunctiva are revealed upon verification of flow through the apparatus, which is indicated by drops forming at the outer end of the tube. If corkscrew vessels are present or if the scleral pocket seems encapsulated, we frequently administer 5-fluoruracil (5-FU) injections into the subconjunctival area during the follow-up period.

### 2.2. Eligibility Criteria

Data of a total of 173 patients who underwent TE and 269 patients who underwent PMS implantation during the time of recruitment were reviewed. Exclusion criteria included an incomplete data set of the initial examinations, inability to return for scheduled protocol visits and an observation period of less than 6 months. In agreement with the guidelines of the World Glaucoma Association, the study only included one eye from each qualified patient [13]. 

### 2.3. Data Collection

Retrospective data collection on age, gender, ethnicity, glaucoma type and prior procedures was performed using digital patient records (Fidus Version 24.12.2, Darmstadt, Germany). The results of perimetric testing, best-corrected visual acuity (BCVA), applanatory pressure measurement, slit lamp examination, number of postoperative 5-FU applications and anti-glaucomatous drug applications were all included in the pre- and postoperative examinations. Following surgery, follow-up checks were scheduled at day 1, month 1, month 3, month 6, month 8 and month 12. In cases when patients visited our clinic more frequently than planned, these visits were taken into account for success rate calculations. In case of an unplanned visit, only the IOP values that were closest to the predefined interval were included in the statistical analysis of IOP evolution. The occurrence of postoperative adverse events, as well as the necessity for revision surgeries, were noted. Bulbar hypotension, defined as an IOP < 5 mmHg, postoperative hyphema, choroidal separation, vitreous hemorrhage or an exposed Tenons capsule at any stage during follow-up were considered adverse events. The frequency of subconjunctival 5-FU injections was recorded; however, these were not considered a revision procedure. The automated Humphrey Visual Field Analyzer II (HFA II, model 750; Carl Zeiss Meditec AG, Jena, Germany) was used for visual field testing (standard program of the 30–2 Swedish interactive threshold algorithm (SITA fast)). 

The results of perimetric testing (Hodapp–Parrish–Anderson classification) [14] were used to assign eyes to disease severity groups (early (0 to −5.99 dB), moderate (−6 to −11.99 dB), severe (≤−12 dB)).

### 2.4. Outcome Measures

The primary outcome was surgical success after 6 months for both the TE and PMS groups. In accordance with the Primary Tube Versus Trabeculectomy Study [15], the primary endpoint after 6 months was defined as either complete success (CS), qualified success (QS) or failure (F). If a patient’s IOP attained values of 6–21 mmHg on two consecutive follow-up visits, with a ≥20% drop in comparison to mean preoperative IOP in both visits, CS was achieved. Patients who met the aforementioned criteria but required additional medical therapy were considered QS. The main outcome was the overall success rate (OS), which was calculated by combining all CS and QS cases. 

Failure was defined as IOP greater than 21 mmHg in any of two consecutive postoperative visits; IOP reduction of less than 20% in any of two consecutive postoperative visits, IOP less than 5 mmHg in any of two consecutive postoperative visits, the need for revision surgery at any time during follow-up or loss of light perception after surgery.

The study’s secondary outcome was the comparison in overall IOP decrease at 6 months after surgery compared to baseline IOP values between the TE and PMS populations and their individual subgroups. 

As a tertiary outcome, we aimed to compare the postoperative care burden within the first year post surgery between both procedures by analyzing and comparing the number of postoperative supplemental antiglaucoma drugs, the number of postoperative adverse events, the frequency of postoperative 5-FU injections and the number of revision surgeries.

### 2.5. Statistical Analysis

Statistical analysis was performed using IBM SPSS Statistics for Windows, Version 28.0 (IBM Corp.: Armonk, NY, USA). The distribution of continuous variables is indicated by the median (first quartile, third quartile). For categorical variables, both absolute and relative frequencies are given.

Multi-group comparisons for categorical variables are carried out using Fisher (e.g., gender) and chi-square tests (e.g., success rates). For continuous variables, the Mann–Whitney U test is used for such comparisons (e.g., age). For paired comparisons (e.g., between different points in time), the Wilcoxon signed rank test is used (e.g., visual acuity). For all these comparisons, *p*-values are given.

It should be noted that our study is primarily exploratory in nature. Thus, we characterize *p*-values < 0.05 as significant, but point out that we do not correct for multiple testing, and these results should therefore be interpreted with caution. Confidence intervals are also given at the 95% level. Missing values are assumed to occur randomly.

## 3. Results

In total, 442 electronic patients were screened. A total of 187 eyes from 187 patients were enrolled in this study, with 114 patients in the PMS group and 73 patients in the TE group.

### 3.1. Baseline Characteristics

There were no significant differences in any of the demographic or ocular baseline features between treatment groups at enrollment (Table 1). 

### 3.2. Previous Operations

Prior procedures included lens surgery, retinal therapies (such as retinal laser coagulation, retinocryotherapy, pars plana vitrectomy and intravitreal injections) and glaucoma surgery (such as TE, PMS, cyclophotocoagulation, i-Stent and selective laser trabeculoplasty). Both TE and PMS showed no significant difference in frequency of previous operations (*p* = 0.26). Additionally, there were no notable variations between TE and PMS in their respective subgroups (p_early glaucoma_ = 0.53; p_moderate glaucoma_ = 1; p_severe glaucoma_ = 0.4; p_POAG_ = 0.59; and p_PEXG_ = 0.47).

### 3.3. Outcomes

There was no discernible difference in OS or CS between the TE study group and the PMS group after 6 months, with the former having an OS rate of 54.1% and the latter having an OS rate of 60% (*p* = 0.67). Furthermore, the overall success rate did not differ significantly among eyes grouped according to severity or glaucoma subtype between the two surgical methods, as shown in Table 2.

### 3.4. Intraocular Pressure

**Figure 1:**
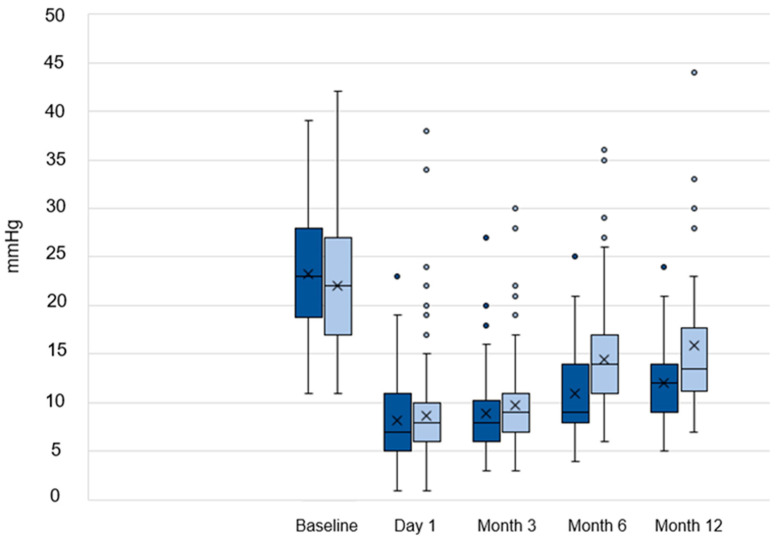
Graphical representation of IOP development throughout follow-up within the TE (dark blue) and PMS groups (light blue). Note that time intervals are not to scale.

At every follow-up period, there was a substantial IOP decrease from baseline in both the TE and PMS groups (*p* < 0.01) as shown in Figure 1. The PMS group showed an average pressure reduction of 7 mmHg, the TE group of 12 mmHg (*p* < 0.01). There was also no significant difference in IOP reduction between the subgroups (*p* < 0.01).

### 3.5. Medical Therapy

As shown in Table 3, both TE and PMS showed a significant reduction in the need for pressure-lowering topical medications. There were no significant differences in the various subgroups.

### 3.6. Postoperative Interventions

On average, 3 (1;4) 5-FU injections were administered in the TE group and 2 (1;4) in the PMS group. There was no significant difference in the frequency of 5-FU administration (*p* = 0.29). In addition, the subgroups also showed no significant differences in the frequency of 5-FU application between the TE and PMS cohorts (p_early_ = 0.71; p_Moderate_ = 0.95; p_Severe_ = 0.51; p_POAG_ = 0.20; p_PEXG_ = 0.67).

### 3.7. Postoperative Complications

Table 4 provides an overview of the postoperative complications in each group. Bulbar hypotonia occurred significantly more often in the TE study group during the first 3 weeks after surgery in comparison to the PMS group (p_7–14d_ < 0.01; p_15–21d_ < 0.01). From 90 days after surgery, however, both groups showed no significant difference in the frequency of persistent bulbar hypotonia (*p*_>90d_ = 0.19). Except for a significantly higher number of choroidal detachments in patients with moderate and severe glaucoma damage (p_moderate_ = 0.02; p_severe_ = 0.02), there were no significant differences in postoperative complications in patients with different glaucoma severity.

### 3.8. Reoperation for Complications

In the study population, as shown in Table 5, there was no significant difference in the number of single revisions (*p* = 0.05), but the time to revision was significantly shorter in the TE group compared to the PMS group (*p* = 0.01). However, a second revision was performed significantly more frequently in the TE group than in the PMS group (*p* < 0.01). In addition, a single revision was significantly more common in the TE group with early glaucomatous disease (*p* = 0.01) (Table 6). There were no relevant differences in terms of revision surgery according to severity or glaucoma genesis.

## 4. Discussion

While TE remains the gold standard in the field of glaucoma surgery, encouraging findings have been shown for the relatively new PMS. Yet, although data are already available, there is still a lack of information on long-term efficacy and safety in real-world settings. Long-term studies comparing the efficacy and safety of TE and PMS in heterogeneous patient populations are, in particular, still rare. 

Our retrospective comparative analysis of the two surgical methods with an OS rate of 54.1% after TE and 60% after PMS implantation, demonstrated no significant difference in surgical success between the two groups (*p* = 0.17). Trials with larger study cohorts had previously shown success rates ranging from 53.9% to 92.3% for CS and from 61.9% to 92.6% for OS for the PMS [16,17,18,19]. The OS rate of the present study is thus slightly lower than the previously published data. This may be due to the fact that, in contrast to earlier studies, this trial was conducted in a heterogeneous patient population.

Previous studies have primarily evaluated the effectiveness of PMS implantation in POAG patients. At the moment, further research investigating the efficacy of PMS in other types of glaucoma is lacking.

In a nonrandomized trial conducted over a six-month period as part of the Dresden Glaucoma and Treatment Trial (DGTS), Pillunat et al. compared PMS and TE in POAG, with 26 patients in each group [18]. The authors describe no statistically significant difference between the two groups in terms of IOP reduction and freedom from medication, but substantially greater rates of postoperative interventions within the TE group [18]. A larger study comparing TE and PMS in relation to POAG was published by Fili et al. The authors conducted a prospective analysis, observing data from 150 eyes in both the TE and PMS groups over a 12-month period [9]. Within this study, PMS was less effective than TE in lowering IOP, although it did reduce the amount of antiglaucoma medications used in comparison to baseline. Additionally, in the treatment of intermediate to advanced POAG, TE provided higher absolute success rates than PMS [8]. Baker et al. prospectively examined a total of 527 patients with POAG (TE: 132; PMS: 395) as part of a multi-center randomized study over one year. Their data also demonstrated lower success rates within the PMS group compared to the TE group (53.9% vs. 72.7%; *p* < 0.01).

Our study is characterized by the fact that in addition to patients with POAG, patients with other types of glaucoma were also included. Although patients with POAG made up approximately 60% of the study cohort (TE: 63.2%; PMS: 61.7%), there was also a high proportion of patients with pseudoexfoliative glaucoma (PEXG) (TE: 18.9%; PMS 19.1%). While an OS of 54.4% after TE was achieved in patients with POAG, an OS of only 35.71% was achieved within the PEXG group. This difference in success rates could also be observed within the PMS group (POAG: 70.8%; PEXG: 45.5%). Although a direct comparison of the subgroups can be limited due to the differences in baseline characteristics, as has been described before [20,21], one of the first studies to compare PMS in POAG and PEXG was conducted by Nobl et al. They showed no significant difference in OS between POAG and PEXG patients [22] despite low-dose MMC intraoperatively (0.2 mg/mL). We have higher failure rates within our subgroups than Nobl et al. [22].

Particularly striking is the relatively high failure rate in the context of PMS surgery in PEXG patients (55%). The differences in success between POAG and PEXG seen in our study are in line with other reports that included subgroup analyses after TE [23]. Schenker et al. described a reduction in the failure rate in the context of intraoperative high-dose MMC therapy (0.4–0.5 mg/mL) [11]. The extent to which a higher dose leads to an improved outcome, particularly in PEX patients, needs to be clarified in further studies.

While there have been numerous reports describing long-term IOP development in TE- and PMS-specific trials, there have only been very few studies comparing both procedures. As previously shown by Pillunat et al., a significant IOP reduction was demonstrated in both groups [18]. In the report by Baker et al., there was a greater IOP drop in the TE group than in the PMS group throughout the first postoperative year (45.4% in the TE group versus 29.1% in the PMS group, *p* < 0.01).

Contrarily, Fili et al., describe no difference in IOP reduction between TE and PMS in their study. Similarly, we did not observe a significant difference between the two groups [9]. This may be due to the significant but less substantial reduction in IOP in our cohort after TE compared to Baker et al. Baseline characteristics did not differ between both cohorts of the present study. Having accounted for disease severity, the distribution of glaucoma subtype and number of previous operations, we do not assume that the results provided in the present study underly any form of major patient selection bias.

In addition to a significant IOP reduction, our data also show a significant reduction in the number of topical pressure-lowering glaucoma medications for both procedures, which is in line with previous reports [9,17,18,19]. Our data also confirm the lower short term complication rate of PMS implantation compared to TE. In particular, postoperative bulbar hypotony and the associated choroidal detachment were significantly less frequent within the first few weeks in patients treated with PMS. Consistent with our findings, hypotonia rates in TE ranged from 14.7% to 49.6% in earlier trials with bigger research populations, but ranged from 12% to 28.9% in the PMS group [9,17,18]. In the present study this difference was particularly evident in advanced glaucoma.

The proposed hypothesis suggesting a potentially challenging surgical scenario within the advanced glaucoma subgroup could not be substantiated. No significant differences were observed in the preoperative profiles, both within the overall cohort (*p* = 0.26) and across varying degrees of severity (*p* = 0.35).

It is important to note that the pre-operative administration of acetazolamide may have impacted postoperative IOP development. Patients received oral acetazolamide preoperatively and discontinued it on the day of surgery, when they were given acetazolamide intravenously (500 mg) for the final time. The possibility remains that acetazolamide influenced patients’ IOP within the first day post-surgery, given its reported half-life of 6–8 h [24]. Nevertheless, IOP values at 1, 6, 12 and 24 months postoperatively are not influenced by prior acetazolamide therapy, providing a clear representation of IOP development post-surgery without systemic pressure-lowering therapy interference.

In the long term, neither the TE or PMS populations nor any of the subgroups showed a statistically significant variation in the number of postoperative complications. The most common surgical intervention in both groups was revision surgery for inadequate drainage cushions (TE: 14.9%; PMS 16.5%). This is in line with previous studies, which indicate a wide range of filter pad revision rates of 4–20.2% in PMS [12,16,18]. Anterior chamber hemorrhage or flattening of the anterior chamber required significantly more frequent flushing of the anterior chamber post TE (10.8%) than post PMS (1.7%). The majority of patients who required such an intervention after TE were patients with PEX glaucoma. It is noteworthy that in contrast to patients with POAG (8.7%), 28.6% of patients with PEX glaucoma required anterior chamber revision after TE. This trend is also evident in the PMS group (POAG: 1.4% vs PEX glaucoma: 9.1%).

The TE cohort’s earlier requirement for revision surgery is confirmed by the significantly shorter time interval until revision surgery. This begs the question of whether PMS implantation tends to have a higher long-term revision rate despite being less expensive and complication-intensive in the short term, as shown in previous studies [16,25].

One of the few groups that has examined data up to five years following the implantation of a PMS is Battle et al. The data presented show that there was minimal postoperative treatment required after the follow-up period of 5 years with an IOP-reduction of 46.7% from baseline. Even though these results are promising, it is vital to keep in mind the small trial size (n = 23), the homogenous patient group consisting solely of patients with POAG and the success rates after one year (100%) that were previously reported but were never recreated [8,26].

It is therefore imperative to close this knowledge gap with larger heterogeneous long-term studies. This should improve the differentiated selection of a suitable surgical procedure in everyday clinical practice for all degrees of severity and subgroups.

### Limitations

Due to its design, this study has some limitations.

First, the retrospective analysis does not provide any meaningful information about the future development of success rates for the two cohorts. To provide an accurate prognosis, additional longitudinal studies with extended follow-up periods are essential. With regards to the cases of failure in patients, further longitudinal studies could be meaningful to better understand postoperative development, especially after revision surgery.

Second, the proportion of patients with pigmentary glaucoma treated with PMS was higher than in the TE cohort. A selection bias cannot be ruled out here. Overall, however, there was no significant difference in the distribution of the glaucoma subgroups in relation to the surgical methods (*p* = 0.22).

The third limitation of our study regards the number and level of training of the operating surgeons. Two experienced senior physicians and glaucoma surgeons operate in our clinic. Comparable studies can include both more and fewer surgeons with different levels of training [15,16,17,18]. A differentiated analysis is difficult due to the limited data and lack of reference values.

Fourth, we have considered most known factors influencing the outcome of glaucoma surgery, such as age, sex, type and severity of glaucoma and previous surgeries; however, postoperative behavior and individual factors may have contributed to the outcome findings. Furthermore, unmeasured confounding factors cannot be ruled out in this retrospective analysis. Further investigation is needed to validate the findings.

## 5. Conclusions

This study compared the six-month success rates of TE and PMS in diverse glaucoma cohorts, thereby providing data on real-life performance of these glaucoma treatment procedures at a time when there is a continuous transition away from TE and towards alternative IOP-lowering treatments. The subgroup-based assessment further refines our understanding in the context of heterogenous glaucoma management, demonstrating that, even though it is a relatively new device, the PMS did not appear inferior to the TE in all subgroups in terms of IOP reduction and freedom from medication to baseline. As surgeons gain more experience with the system’s implantation, future long-term outcomes may change.

## Figures and Tables

**Table 1:** Basic characteristics of study population. n = number; * = median (25th percentile; 75th percentile); M = male; F = female, R = right eye; L = left eye; POAG = primary open-angle glaucoma; PEXG = pseudoexfoliative glaucoma; MD = mean deviation; dB = decibel; PDS = pattern standard deviation; logMAR = logarithm of the minimum angle of resolution; % = percentage; PCL = posterior chamber lens; ** Mann–Whitney U test; *** Fisher test; **** Chi-square test; ***** Wilcoxon test; *p* < 0.05 are marked in bold.

Characteristics	Trabeculectomy	Preserflo MicroShunt	*p*-Value
Eyes (n)	73	114	
Patients (n)	73	114	
Age (years) *	68 (58;77)	69 (61;77)	0.81 **
Gender (F:M)	44:29	58:56	0.18 ***
Study eye (R:L)	36:37	62:52	0.55 ***
Eyes (n) according to type of glaucoma			
(a) POAG	46 (63.2%)	71 (61.7%)	0.22 ****
(b) PEXG	14 (18.9%)	22 (19.1%)	
(c) Normal-tension glaucoma	1 (1.4%)	2 (1.7%)	
(d) Pigmentary glaucoma	1 (1.4%)	7 (6.1%)	
(e) Other secondary glaucoma	6 (8.1%)	11 (9.65%)	
(f) Ocular hypertension	0	1 (0.9%)	
(g) Aphakic glaucoma	2 (2.7%)	0	
(h) Primary angle closure glaucoma	2 (2.7%)	0	
MD (dB) *	13.39 (6.64;20.69)	12.5 (4.74;22.19)	0.70 **
PDS *	8.06 (4.48;10.32)	7.57 (3.55;10.24)	0.96 **
Visual acuity (logMAR) *			
(a) preoperative	0.3 (0.1;0.55)	0.2 (0.1;0.4)	TE: 0.53 *****
(b) one month postoperative	0.2 (0.1;0.45)	0.2 (0.1;0.4)	PMS: 0.62 *****
Severity (n,%)			
(a) Early	15 (20.3%)	34 (29.6%)	0.11 ****
(b) Moderate	19 (25.7%)	17 (14.8%)	
(c) Severe	39 (52.7%)	63 (54.8%)	
Lens status (n,%)			
(a) aphak	2 (2.7%)	1 (0.9%)	0.57 ****
(b) phak	31 (41.9%)	46 (40%)	
(c) pseudophak	40 (54.1%)	67 (58.3%)	
Combined operation (n,%)			
(a) with Phako/PCL	8 (10.8%)	12 (10.4%)	1 ***
(b) without Phako/PCL	65 (87.8%)	102 (88.7%)	

**Table 2:** Outcome rates for the entire study population and according to disease severity groups and type of glaucoma. n = number; % = percentage; POAG = primary open-angle glaucoma; PEXG = pseudoexfoliative glaucoma; **** chi-square test; *p* < 0.05 are marked in bold.

	Trabeculectomy				Preserflo MicroShunt				*p*-Value (Overall Success)
	Overall Success	Complete Success	Qualified Success	Failure	Overall Success	Complete Success	Qualified Success	Failure	
Total study population (n, %)	40 (54.1%)	6 (8.1%)	34 (46%)	33 (44.6%)	69 (60%)	23 (20%)	46 (40%)	45 (39.1%)	0.67 ****
Disease severity groups (n, %)									
(a) Early	10 (66.7%)	1 (6.7%)	9 (60%)	5 (33.3%)	20 (58.8%)	4 (11.8%)	16 (47.1%)	14 (41.1%)	0.8 ****
(b) Moderate	9 (47.4%)	0	9 (47.4%)	10 (52.6%)	10 (58.8%)	3 (17.7%)	7 (41.2%)	7 (41.2%)	0.7 ****
(c) Severe	21 (53.9%)	5 (12.8%)	16 (41%)	18 (46.2%)	39 (61.9%)	16 (25.4%)	23 (36.5%)	24 (38.1%)	0.68 ****
Type of glaucoma (n, %)									
(a) POAG	25 (54.4%)	4 (8.7%)	21 (45.7%)	21 (45.7%)	50 (70.8%)	16 (22.5%)	34 (47.9%)	21 (29.2%)	0.4 ****
(b) PEXG	5 (35.7%)	0	5 (35.7%)	9 (64.3%)	10 (45.5%)	2 (9.1%)	8 (36.4%)	12 (54.6%)	0.71 ****

**Table 3:** Significance of the reduction in the number of IOP-modifying medication (*p*-value) within the TE and PMS study group, as well as for subgroups according to disease severity and type of glaucoma. * = median (25th percentile; 75th percentile); POAG = primary open-angle glaucoma; PEXG = pseudoexfoliative glaucoma; ** Mann–Whitney U test; *p* < 0.05 are marked in bold.

	Trabeculectomy		*p*-Value	PreserfloMicroShunt		*p*-Value
	Baseline *	12 Month Post Surgery *		Baseline *	12 Month Post Surgery *	
Total study population (n, %)	4 (3;4)	0 (0;0)	<0.01	3 (3;4)	0 (0;2)	**<0.01** **
Disease severity groups (n, %)						
(a) Early	4 (3;4)	0 (0;0)	<0.01	3 (3;4)	1 (0;3)	**<0.01** **
(b) Moderate	4 (3;4)	0 (0;0)	<0.01	3 (3;4)	0 (0;2)	**<0.01** **
(c) Severe	4 (3;4)	0 (0;0)	<0.01	3 (2;4)	0 (0;2)	**<0.01** **
Type of glaucoma (n, %)						
(a) POAG	4 (3;4)	0 (0;0)	<0.01	3 (3;4)	0 (0;2)	**<0.01** **
(b) PEXG	4 (3.25;4)	0 (0;0)	<0.01	3 (2;4)	0 (0;2)	**<0.01** **

**Table 4:** IOP-reduction for the entire study population of TE and PMS study groups, as well as for subgroups according to type of glaucoma. n = number; % = percentage; POAG = primary open-angle glaucoma; PEXG = pseudoexfoliative glaucoma; *** Fisher test; *p* < 0.05 are marked in bold.

	Total Study Population			Type of Glaucoma					
Complications			*p*-Value	POAG		*p*-Value	PEXG		*p*-Value
	Trabeculectomy	Preserflo MicroShunt		Trabeculectomy	PreserfloMicroShunt		Trabeculectomy	PreserfloMicroShunt	
erall (n,%)	56 (75.7%)	91 (79.1%)	0.59 ***	37 (80.4%)	55 (77.5%)	0.82 ***	11 (78.6%)	19 (86.4%)	0.66 ***
bulbar hypotonia (n,%)	43 (58.1%)	65 (56.5%)	0.88 ***	27 (58.7%)	38 (53.5%)	0.70 ***	11 (78.6%)	13 (29.1%)	**<0.01** ***
(a) 7–14 d (n,%)	29 (39.2%)	7 (6.1%)	<0.01 ***	18 (39.1%)	4 (5.6%)	0 ***	9 (64.3%)	2 (9.1%)	**<0.01** ***
(b) 15–21 d (n,%)	15 (20.3%)	3 (2.6%)	<0.01 ***	10 (21.7%)	2 (2.8%)	**<0.01** ***	4 (28.6%)	1 (4.6%)	0.06 ***
(c) >90 d (n,%)	6 (8.1%)	4 (3.5%)	0.19 ***	5 (10.9%)	2 (2.8%)	0.11 ***	1 (7.1%)	0	0.39 ***
hyphema (n,%)	18 (24.3%)	26 (22.6%)	0.86 ***	13 (28.3%)	18 (25.4%)	0.83 ***	4 (28.6%)	5 (22.7%)	0.71 ***
amotio choroideae (n,%)	28 (37.8%)	16 (13.2%)	<0.01 ***	19 (41.3%)	9 (12.7%)	**0.02** ***	7 (15%)	4 (18.2%)	0.07 ***
vitreous hemorrhage (n,%)	5 (6.8%)	0	<0.01 ***	3 (6.5%)	0	0.07 ***	1 (7.1%)	0	0.39 ***
exposed tenon’s capsule (n,%)	0	0	1 ***	0	0	1 ***	0 (%)	0	1 ***

**Table 5:** Overview revision surgery for the entire study population; n = number; % = percentage; * = median (25th percentile; 75th percentile); POAG = primary open-angle glaucoma; PEXG = pseudoexfoliative glaucoma; ** Mann–Whitney U test; *** Fisher test; *p* < 0.05 are marked in bold.

	Total Study Population		
Revisions-OP			*p*-Value
	Trabeculectomy	Preserflo MicroShunt	
(a) one-off (n,%)	29 (39.2%)	29 (25.2%)	0.05 ***
(b) twice (n,%)	9 (12.2%)	0	0.00 ***
(c) three times (n,%)	1 (1.4%)	1 (0.9%)	1 ***
time interval to the initial Operation *	13.5 (3;96.3)	163 (20;248.5)	0.00 **
(1) Revision-surgery (n,%)	11 (14.9%)	19 (16.5%)	0.84 ***
(2) Flushing of the ant. Chamber (n,%)	8 (10.8%)	2 (1.7%)	0.01 ***
(3) Suturlyse (n,%)	10 (13.5%)	/	/
(4) (Re-)Trabeculoplasty (n,%)	2 (2.7%)	6 (5.2%)	0.48 ***
(5) Preserflo (n,%)	6 (8.1%)	/	/
(6) Pars plana vitrectomy (n,%)	5 (6.8%)	2 (1.7%)	0.11 ***
(7) Cyclophotocoagulation (n,%)	1 (1.4%)	5 (4.4%)	0.41 ***
(8) Paul-Implantat (n,%)	/	3 (2.6%)	/

**Table 6:** Overview revision surgery according type of glaucoma; n = number; % = percentage; * = median (25th percentile; 75th percentile); POAG = primary open-angle glaucoma; PEXG = pseudoexfoliative glaucoma; ** Mann–Whitney U test; *** Fisher test; *p* < 0.05 are marked in bold.

	Type of Glaucoma					
Revisions-OP	POAG		*p*-Value	PEX Glaucoma		*p*-Value
	Trabeculectomy	Preserflo MicroShunt		Trabeculectomy	Preserflo MicroShunt	
(a) one-off (n,%)	15 (32.6%)	16 (22.5%)	0.28 ***	7 (15%)	8 (36.4%)	0.5 ***
(b) twice (n,%)	6 (13.1%)	0	0.00 ***	2 (14.3%)	0	0.14 ***
(c) three times (n,%)	1 (2.2%)	0	0.39 ***	0	1 (4.6%)	1 ***
time interval in days to the initial revision operation *	12 (3.5;86.5)	167 (35;224.5)	**0.01** **	11 (7;7)	121 (14;199)	0.16 **
(1) Revision-surgery (n,%)	9 (19.6%)	13 (18.3%)	1 ***	2 (14.3%)	5 (22.7%)	0.68 ***
(2) Flushing of the ant. Chamber (n,%)	4 (8.7%)	1 (1.4%)	0.16 ***	4 (28.6%)	2 (9.1%)	0.38 ***
(3) Suturlyse (n,%)	6 (13.1%)	/	/	2 (14.3%)	/	/
(4) (Re-)Trabeculoplasty (n,%)	0	3 (4.2%)	0.28 ***	1 (7.1%)	2 (9.1%)	1 ***
(5) Preserflo (n,%)	0	0	1 ***	0	0	1 ***
(6) Pars plana vitrectomy (n,%)	2 (4.4%)	1 (1.4%)	0.56 ***	2 (14.3%)	1 (4.6%)	0.56 ***
(7) Cyclophotocoagulation (n,%)	1 (2.2%)	1 (1.4%)	1 ***	0	2 (9.1%)	0.51 ***
(8) Paul-Implantat (n,%)	0	2 (2.8%)	0.52 ***	0	0	1 ***

## Data Availability

The original contributions presented in the study are included in the article, further inquiries can be directed to the corresponding authors.
